# Cortical Grey Matter and Subcortical White Matter Brain Microstructural Changes in Schizophrenia Are Localised and Age Independent: A Case-Control Diffusion Tensor Imaging Study

**DOI:** 10.1371/journal.pone.0075115

**Published:** 2013-10-04

**Authors:** Chiara Chiapponi, Fabrizio Piras, Federica Piras, Sabrina Fagioli, Carlo Caltagirone, Gianfranco Spalletta

**Affiliations:** 1 Laboratory of Clinical and Behavioural Neurology, IRCCS Santa Lucia Foundation, Rome, Italy; 2 Department of Neuroscience, “Tor Vergata” University, Rome, Italy; University G. D’Annunzio, Italy

## Abstract

It is still unknown whether the structural brain impairments that characterize schizophrenia (SZ) worsen during the lifetime. Here, we aimed to describe age-related microstructural brain changes in cortical grey matter and subcortical white matter of patients affected by SZ. In this diffusion tensor imaging study, we included 69 patients diagnosed with SZ and 69 healthy control (HC) subjects, age and gender matched. We carried out analyses of covariance, with diagnosis as fixed factor and brain diffusion-related parameters as dependent variables, and controlled for the effect of education. White matter fractional anisotropy decreased in the entire age range spanned (18–65 years) in both SZ and HC and was significantly lower in younger patients with SZ, with no interaction (age by diagnosis) effect in fiber tracts including corpus callosum, corona radiata, thalamic radiations and external capsule. Also, grey matter mean diffusivity increased in the entire age range in both SZ and HC and was significantly higher in younger patients, with no age by diagnosis interaction in the left frontal operculum cortex, left insula and left planum polare and in the right temporal pole and right intracalcarine cortex. In individuals with SZ we found that localized brain cortical and white matter subcortical microstructural impairments appear early in life but do not worsen in the 18–65 year age range.

## Introduction

It is essential to characterize the pathogenesis of schizophrenia (SZ) and evolution of the illness during the lifespan to develop future therapeutic strategies and improve disease outcomes.

According to the classical neurodevelopmental hypothesis of SZ pathogenesis, genetic and environmental factors can interfere with the development of the central nervous system, leading to permanent errors in the organization of axonal connections or other complex morphological characteristics of the brain as cell patterning or brain asymmetry [1]–[4]. The possibility of knowing when specific brain structures are damaged in SZ, whether the damage is static, whether it worsens or it diminishes over time, and the possibility of predicting age-related trajectories of brain structures would be a turning point in caring for these patients. Although many efforts have been made to clarify this issue, the complexity and multifactorial features of SZ make a complete understanding still far [5]–[7].

Brain abnormalities related to SZ can be profitably investigated using magnetic resonance imaging (MRI). In particular, we can look at age-related trajectories followed by diffusion tensor imaging (DTI) parameters. These microstructural indexes are reliable and sensitive tools for detecting subtle, but critical, structural abnormalities even where macrostructural volumetric impairments cannot be observed. Due to its properties, DTI provides significant descriptions of age-related changes in neural development. In particular, it helps to explain mechanisms of physiological and pathological aging [8]–[12] as well as different neuropsychiatric conditions [13]–[15] and it also allows to map brain plasticity, even in elderly subjects [16]. As DTI is sensitive to the intrinsic properties of water diffusion, it has been used to obtain information about fine tissue characteristics through two quantitative parameters: i) fractional anisotropy (FA) [17], [18], and ii) diffusivity [19]. The latter can be expressed through three different measures: i) axial diffusivity (AD) which represents diffusion along fibre direction [20], [21], ii) radial diffusivity (RD), perpendicular to fibre direction [22], and iii) mean diffusivity (MD) which describes the rotationally invariant magnitude of water diffusion [23]. Notably, diffusion related parameters have been reported to be less sensitive to image noise than FA [24], giving them a key role in DTI studies [25]–[28]. Thus, only the concurrent investigation of interplaying diffusivity- and anisotropy-related parameters can help clarifying brain microstructural features in SZ and other neuropsychiatric disorders.

The physiological age-related trajectories of brain microstructural parameters have been widely investigated. Studies in healthy aging individuals agree that a decrease in white matter (WM) FA occurs in late adulthood, mainly in prefrontal, temporal and parietal brain areas [29], [30]. These are the last brain regions myelinated during neurodevelopment and they are also the most vulnerable to myelin breakdown during normal aging [31]. Investigation of RD and AD in WM fibres is crucial to better understand the different mechanisms underlying WM age-related changes [32]. Indeed, processes such as demyelination of axons [33], subtle disruption of myelin sheaths [34], [35] and reduction of myelinated fibre length [34] have been reported in normal aging, and RD and AD can differentially reflect these phenomena. With regard to grey matter (GM), comparisons between MD of healthy elderly and young subjects revealed higher cortical MD in the elderly group [36], [37], and increased diffusivity with age has been related to the loss of normal tissue microstructure, such as disruption of neuronal cell membrane that restrict Brownian motion of water molecules [19].

In MRI studies focusing on how SZ affects brain microstructure, varied and sometimes discordant findings have been obtained [20]. Microstructural differences between SZ patients and healthy control (HC) subjects have been mainly investigated by comparing FA in WM tracts. According to a recent meta-analysis [38], areas characterized by FA reductions in SZ include: i) the deep left frontal WM, which is traversed by WM tracts interconnecting the frontal lobe, thalamus and cingulate gyrus, and ii) the left temporal deep WM, which is traversed by WM tracts interconnecting the frontal lobe, insula, hippocampus–amygdala, temporal and occipital lobes.

Nevertheless, many intriguing and controversial questions still remain: 1) which structural brain changes are precocious? 2) Are these processes static in time or do they progress after illness onset inducing pathological age-related changes in brain structure? 3) Do brain abnormalities in patients depend on age or on age-related factors such as illness duration [1]? 4) Is the magnitude of age-related brain structural changes reported for young SZ patients in MRI studies linear during the lifespan [39]?

Several studies that focused on brain microstructure through DTI and measured the relationship between FA and age in SZ reported contradictory findings [40]–[43]. Moreover, a complete exploration of all the interplaying DTI-related parameters is lacking in the literature. Thus, the aim of the present study was to investigate the age-related changes of strategic brain DTI indices in SZ and to compare their trajectories with those of HC. In particular, we considered FA, AD and RD to investigate WM and MD to investigate cortical GM.

## Materials and Methods

### Participants

We assessed 69 patients with a diagnosis of SZ according to the Diagnostic and Statistical Manual of Mental Disorders IV-Edition, text revised [44] at the IRCCS Santa Lucia Foundation in Rome. The clinician who had been treating the patients and knew their clinical history, but who was blind to the aims of the study, made the preliminary diagnosis. Then a senior research psychiatrist confirmed all preliminary diagnoses using the Structured Clinical Interview for DSM-IV-TR-Patient Edition (SCID-I/P) [45]. If the clinicians disagreed, more data were gathered and the diagnostic process continued until a final consensus diagnosis was reached. If no agreement could be reached, the patient was removed from the sample.

Inclusion criteria were: 1) age between 18 and 65 years, 2) at least five years of education, and 3) suitability for MRI scanning. Exclusion criteria were: 1) history of alcohol or drug abuse in the two years before the assessment, 2) lifetime drug dependence, 3) traumatic head injury with loss of consciousness, 4) past or present major medical illness or neurological disorders, 5) any additional psychiatric disorders or mental retardation, 6) any potential brain abnormalities and microvascular lesions as apparent on conventional T2- and FLAIR-scans; in particular, the presence, severity, and location of vascular lesions were rated by two experts according to a protocol designed for the Rotterdam Scan Study [46]. Generally, they were considered present in cases of hyperintense lesions on both proton-density and T2-weighted (see image acquisition) and rated semiquantitatively as 0 (none), 1 (pencil-thin lining), 2 (smooth halo), or 3 (large confluent) for three separate regions; adjacent to frontal horns (frontal caps), adjacent to the wall of the lateral ventricles (bands), and adjacent to the occipital horns (occipital caps). The total vascular lesion load was calculated by adding the region-specific scores (range, 0–9). In the present study, only participants rated 0–1 were included, 7) dementia or cognitive deterioration according to DSM-IV-TR criteria and Mini-Mental State Examination (MMSE) [47] score lower than 25, consistent with normative data in the Italian population [48].

Overall severity of SZ symptoms was assessed using the Positive and Negative Syndrome Scale (PANSS) [49], which is a 30-items seven-point rating instrument that yields scores of positive (7 items, with a total score ranging from 7 to 49) and negative (7 items, with a total score ranging from 7 to 49) symptoms, and of general psychopathology (16 items, with a total score ranging from 16 to 112). Age at onset was defined as age at onset of positive or negative symptoms preceding the first hospitalization, which was investigated in an interview with patients and first-degree relatives.

All patients were receiving stable oral dosages of one or more atypical antipsychotics such as risperidone, quetiapine, and olanzapine. Antipsychotic dosages were converted to equivalents of olanzapine [50].

We recruited 69 HC in the same geographical area. They were carefully matched, one by one, with the patients for age and gender. All HC were screened for a current or lifetime history of DSM-IV-TR Axis I and II disorders using the SCID-I [51] and SCID-II [52]; they were also assessed to confirm that no first-degree relative had a history of psychosis.

Sociodemographical and clinical characteristics of the HC and SZ samples are shown in Table 1.

**Table 1:** Sociodemographic and clinical characteristics of 69 SZ and 69 HC subjects.

*Characteristics*	*HC (n = 69)*	*SZ (n = 69)*	*t or x^2^*	*df*	*p*
Age (years ± S.D.)	38.09±12.09	38.45±11.70	−0.18	136	0.86
Males, *n* (%)	47 (68)	47 (68)	0.00	1	>0.999
Educational Level(years ± S.D.)	15.06±2.92	12.14±3.17	5.61	136	<0.001
Olanzapineequivalents(mg/day)	–	18.65±17.76	–	–	–
PANSS positive	–	22.87±5.94	–	–	–
PANSS negative	–	20.10±7.35	–	–	–
PANSS generalpsychopathology	–	48.28±12.08	–	–	–

PANSS = positive and negative syndrome scale; S.D. = standard deviation; df = degrees of freedom; HC = Healthy Control; SZ = Schizophrenia.

The study was approved and undertaken in accordance with the guidelines of the Santa Lucia Foundation Ethics Committee. All participants gave their written informed consent to participate in the research after they had received a complete explanation of the study procedures.

### Image Acquisition and Processing

All 138 participants underwent the same imaging protocol, which included 3D T1-weighted, DTI, T2-weighted and FLAIR sequences, using a 3T Allegra MR imager (Siemens, Erlangen, Germany) with a standard quadrature head coil. Whole-brain T1-weighted images were obtained in the sagittal plane using a modified driven equilibrium Fourier transform sequence (TE/TR = 2.4/7.92 ms, flip angle 15°, voxel size 1×1×1 mm^3^) (MDEFT). Diffusion-weighted volumes were acquired using spin-echo EPI (TE/TR = 89/8500 ms, bandwidth = 2126 Hz/vx; matrix size 128×128; 80 axial slices, voxel size 1.8×1.8×1.8 mm^3^) with 30 isotropically distributed orientations for the diffusion sensitising gradients at a b-value of 1000 s/mm^2^ and 2 no diffusion weighted images (b0). Scanning was repeated three times to increase the signal-to-noise ratio [8]. T2 and FLAIR sequences were acquired to screen for brain pathology. Images were processed using FSL 4.1 software (www.fmrib.ox.ac.uk/fsl/). DTI images were corrected for the distortion induced by eddy currents and head motions, by applying a 3D full affine alignment of each image to the mean b0 image.

After distortion corrections, DTI data were averaged and concatenated into 31 (1 b0+30 b1000) volumes. A diffusion tensor model was fitted at each voxel, generating FA, AD (first eigenvalue of the diffusion tensor), RD (average of the second and third eigenvalues) and MD maps.

FA, RD, and AD were assessed in WM tracts, because the diffusion is inherently directional there.

We used Tract-Based Spatial Statistic (TBSS) [53] version 1.2, part of FSL for the post processing and analysis of FA, RD and AD maps in WM. The key features of TBSS overcome the alignment problems [54], [55] and smoothing issues [56] related to conventional VBM-style whole brain approaches for multi-subject DTI images. Briefly, TBSS first projects all subjects’ FA, RD and AD data onto an alignment invariant tract representation, i.e. the skeleton, by means of the nonlinear registration tool FNIRT [57], [58], which uses a b-spline representation of the registration warp field [59]. This process of projecting individual maps onto a mean skeleton helps confining the effect of cross-spatial subject variability that remains after classical non-linear registration. The resulting data are then fed into voxel-wise cross-subject statistics [60].

MD maps were registered to the brain-extracted whole-brain template in Montreal Neurological Institute (MNI) space (http://www.mni.mcgill.ca/) through the FMRIB’s Linear Image Registration Tool (FLIRT) [61]–[63] using the coefficients file obtained by the FA registration as starting guess.

MD was chosen as the parameter to probe GM microstructure because GM tissue lacks directional diffusion. MD of SZ patients and HC were compared within cortical GM regions of interest (ROIs). ROIs selection was performed using the Harvard-Oxford Atlas implemented in FSL. This probabilistic atlas contains 48 cortical and 21 subcortical structural areas, derived by the individual segmentation of T1-weighted images of 21 healthy male and 16 healthy female subjects (ages 18–50), which was carried out by the Harvard Center for Morphometric Analysis. For the purpose of this study, we considered the 48 cortical regions of the atlas distributed as follow: 12 ROIs in the frontal lobe, 16 in the temporal lobe, the insula was a single region, 12 ROIs in the parietal lobe and 7 to the occipital lobe (see Table S1 for the complete list). The ROIs definition from the probabilistic areas was performed with particular care to avoid superimposing adjacent zones; the extracted regions were visually assessed by a trained neuroanatomist to avoid misregistration errors or wrong ROIs identification. Several steps were taken to minimize the risk of partial volume artefacts due to the impact of CSF contamination on MD maps, which was particularly problematic at the interfaces of tissue with CSF-filled spaces [64]. To avoid partial volume effects between cortex and CSF, the MD images were first thresholded to remove all MD values above 3•10^−3^ mm^2^/s, which is the threshold that denotes CSF contribution. Then, when the average MD value was calculated for each cortical ROI in MNI space, care was taken not to include voxels where MD intensity was equal to zero.

### Statistical Analyses

Comparisons between the two diagnostic groups (i.e. SZ and HC) on sociodemographic characteristics (age, gender and educational level) were performed using the t-test or chi-square test.

We chose analysis of covariance (ANCOVA) to compare the diagnostic group age-related changes of brain microstructural MRI indices, evaluated in GM and WM. For both tissues, the DTI parameters were, in turn, the dependent variable, diagnosis was included as fixed factor (independent variable) and age as covariate. We first verified the linear dependence on age of each DTI parameter, the primary hypothesis of ANCOVA. Then, we verified the ANCOVA assumption of homogeneity of regression slopes. Where this hypothesis was fulfilled, we looked for the presence of a main effect of the diagnosis. Years of formal education were included as covariate of no interest to reduce its potential impact on the age results. We did not include illness duration in the model since it resulted strongly collinear with age.

#### Voxel-wise comparison between age-related trajectories of WM parameters in SZ and HC

For the voxel-wise comparison of age-related trajectories of FA, AD and RD on the WM skeleton in SZ and HC, we used a permutation-based approach, namely, the randomise command in the FSL package [60]. The Threshold-Free Cluster Enhancement option [65] was used in the randomise command to obtain the significant differences between groups at p<0.05, after accounting for multiple comparisons by controlling for family-wise error (FWE) rate [66] to avoid false positive results (type I errors).

#### ROI-based comparison between age-related trajectories of cortical GM parameters in SZ and HC

The ROI-based comparison of age-related changes of MD in the cortical GM in SZ and HC was performed using Matlab (vers. 7.1, the MathWorks). For each lobe, we first performed a linear regression of MD as a function of age in each ROI. Thus, we obtained Pearson’s correlation coefficient (r), significance threshold (*p_lin_*), angular coefficient (*m*), and intercept (*MD_0_*). The assumption of homogeneity of regression slopes was verified by comparing angular coefficients through t-tests. In these comparisons, the significance threshold (*p_slope_*), was chosen as a function of the number of ROIs (*N*), examined in each lobe: *p_slope_*<0.05/*N,* where *N* = 12 in the frontal and parietal lobes (*p_slope_*<0.004), *N* = 16 in the temporal lobe (*p_slope_*<0.003), *N* = 1 in the insula (*p_slope_*<0.05), and *N* = 7 in the occipital lobe (*p_slope_*<0.007). For each lobe, in the ROIs where the regression slopes where homogeneous in the two diagnostic groups, we verified the presence of a main effect of diagnosis, comparing *MD_0_* through t-tests. The significance threshold (*p_main_effect_*) was chosen according to the number of ROIs considered in each lobe, i.e. in the frontal lobe *p_main_effect_* = 0.05/9 = 0.0056, in the temporal lobe *p_main_effect_* = 0.05/3 = 0.017 and in the insula, parietal and occipital lobe *p_main_effect_* = 0.05.

## Results

As expected from the matching procedure, the group of SZ patients and that of HC did not significantly differ for age or gender (see Table 1). However, the two groups differed for educational level, and this variable was included in each analysis as covariate of no interest to control for its effect on the results.

### WM Analysis

#### Fractional anisotropy age-related changes

**Figure 1:**
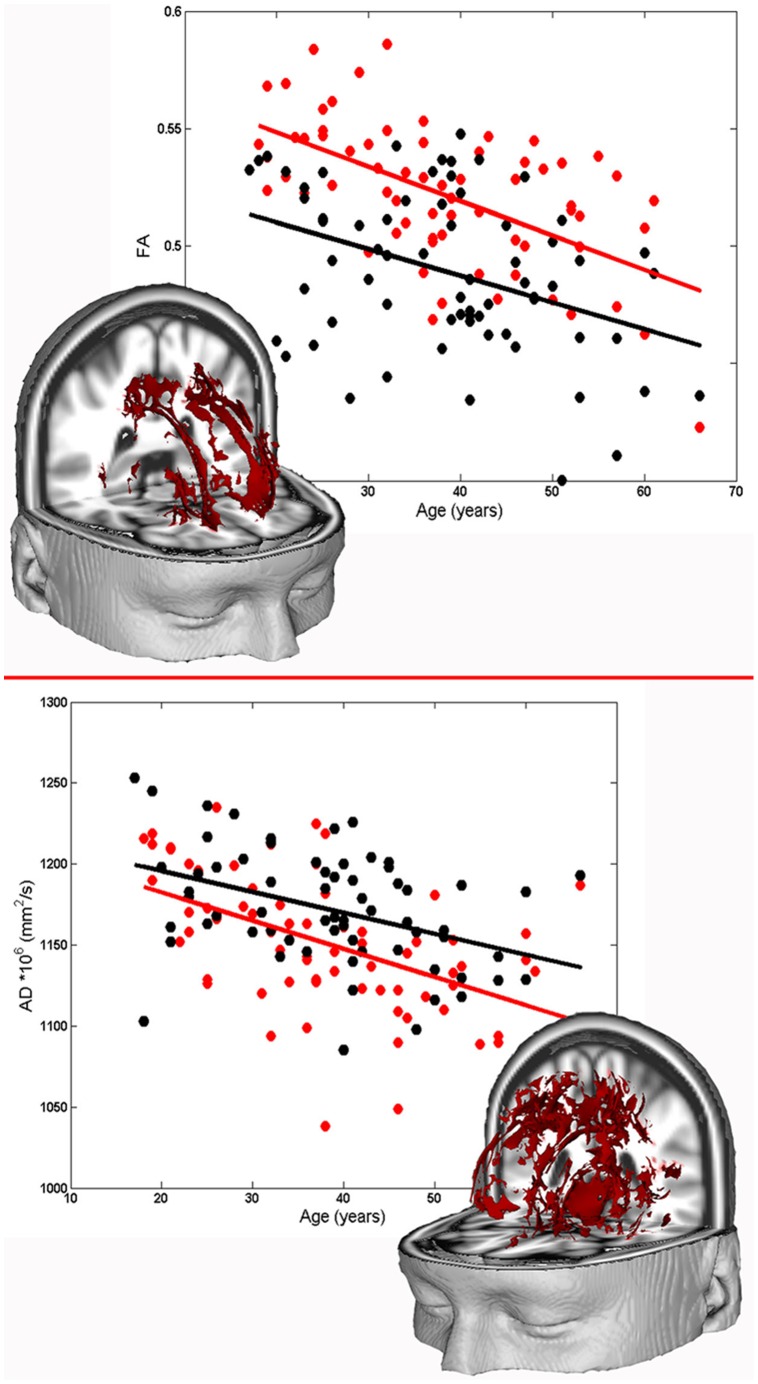
Age-related trajectories of WM DTI parameters of SZ and HC subjects. Upper part: three-dimensional representation of clusters in which a main effect of group characterizes the relationship between FA and age. The scatter plot shows the mean FA value in these clusters as a function of age for SZ (white circles) and HC (red circles). Solid lines represent the linear fits for each group. Lower part: three-dimensional representation of clusters where a significant correlation between AD and age results in both SZ and HC groups. The plot shows the mean AD value in these clusters as a function of age for SZ (white circles) and HC (red circles). Solid lines represent the linear fits for each group.

In the voxel-wise analysis of the age-related changes of WM FA in SZ and HC, a negative correlation between FA and age was found for both diagnostic groups in the entire skeleton. The assumption of homogeneity of regression slopes was fulfilled (i.e. no significant diagnosis by age interaction was found) and a main effect of the diagnosis emerged in a bilateral portion of the WM skeleton mainly located in the corona radiata, corpus callosum, thalamic radiations and external capsule (cluster p-value<0.05, FWE corrected). (see Figure 1).

Coordinates of the statistical peaks in the resulting WM clusters are shown in Table 2.

**Table 2:** White matter clusters where a main effect of diagnosis emerged in fractional anisotropy.

White matterTract	Side	MNI coordinates			Cluster size (mm^3^)
		X	Y	Z	
Superior CR	L	−23	−20	37	6744
Superior CR	R	23	−18	36	5628
Posterior ThR	R	35	−54	15	392
ExC	R	35	−8	3	151
Sagittal stratum	R	40	−28	−4	127

Cluster p-value<0.05, FWE corrected.

L = left; R = right; CR = Corona Radiata; ThR = Thalamic Radiations; ExC = External Capsule.

The reported MNI coordinates identify the voxel of the resulting cluster with the strongest statistics.

#### Radial diffusivity and axial diffusivity age-related changes

Analysis of the age-related trajectories of WM RD in SZ and HC showed no significant correlation between RD and age.

Analysis of WM AD revealed a significant negative correlation between AD and age in a large region of the WM skeleton including, bilaterally, the cingulum, corpus callosum and external and internal capsule (see Figure 1). However, neither a main effect of diagnostic group nor an age by diagnosis interaction emerged, i.e. the regression lines of AD as a function of age for SZ and HC had the same slope and the same intercept. Table 3 summarizes the details describing WM clusters where AD is linearly dependent on age with the same linear relationship in the two diagnostic groups.

**Table 3:** Relationship between axial diffusivity and age in SZ.

White matter tract	Side	MNI coordinates			Cluster size (mm^3^)
		X	Y	Z	
Cortico spinal tract	L	−7	−25	−34	19196
Superior CR	R	21	−25	39	8420
InC	R	28	−17	−3	1148

Cluster p-value<0.05, FWE corrected.

L = left; R = right; CR = Corona Radiata; InC = Internal Capsule.

The reported MNI coordinates identify the voxels with the strongest statistics.

### GM Analysis

Analysis of the linear relationship between MD and age in cortical GM of SZ and HC revealed a significant positive correlation between MD and age in both diagnostic groups in the ROIs shown in Table 4.

**Table 4:** Relationships between mean diffusivity and age in SZ and HC subjects.

ROI	Side	HC				SZ			
		r	p_lin_	m	MD_0_	r	p_lin_	m	MD_0_
**Frontal lobe**									
Frontal operculum cortex	L	0.38	0.001	3.56	747.4	0.43	<0.001	6.00	774.2
Frontal pole	L	0.48	<0.001	6.43	806.3	0.37	0.002	4.92	881.5
	R	0.54	<0.001	6.67	772.1	0.42	<0.001	4.99	856.4
Inferior frontal gyrus, pars opercularis	L	0.57	<0.001	8.01	824.8	0.35	0.003	5.56	983.8
	R	0.60	<0.001	8.65	769.0	0.40	<0.001	5.96	919.5
Middle frontal gyrus	L	0.57	<0.001	9.05	758.1	0.44	<0.001	5.91	885.6
	R	0.55	<0.001	8.70	785.0	0.38	0.001	4.78	964.4
Superior frontal gyrus	L	0.58	<0.001	13.6	852.0	0.34	0.004	6.91	1077.9
	R	0.54	<0.001	13.0	854.4	0.37	0.002	7.14	1084.0
**Temporal lobe**									
Planum polare	L	0.36	0.002	6.97	853.3	0.40	<0.001	12.03	793.4
Superior temporal gyrus, anterior division	R	0.40	<0.001	4.57	817.9	0.38	0.001	4.41	837.2
Temporal pole	R	0.46	<0.001	4.82	815.8	0.36	0.002	5.62	858.4
**Insula**									
Insular cortex	L	0.39	<0.001	3.09	944.3	0.27	0.02	3.91	1027.6
**Parietal lobe**									
Precentral gyrus	L	0.54	<0.001	8.05	876.3	0.35	0.003	4.26	1015.7
**Occipital lobe**									
Intracalcarine cortex	R	0.53	<0.001	3.40	822.6	0.37	0.002	2.53	917.1

The significance threshold, p_lin_, was chosen as a function of the number of ROIs in each lobe. Frontal lobe: p_lin_<0.05/12; Temporal lobe: p_lin_<0.05/16; Insula: p_lin_<0.05; Parietal lobe: p_lin_<0.05/12; Occipital lobe: p_lin_<0.05/7. L = left; R = right; r = Pearson’s correlation coefficient; m = regression angular coefficient; MD_0_ = regression intercept; HC = Healthy Controls; SZ = Schizophrenia; ROI = Region of Interest; MD = Mean Diffusivity.

The ROIs in which MD was significantly correlated with age in the frontal lobe were the left frontal operculum cortex and, bilaterally, the frontal pole and the inferior, middle and superior frontal gyri; the ROIs revealed in the temporal lobe were the left planum polare, right superior temporal gyrus and right temporal pole; the remaining ROIs were the left insula, left precentral gyrus in the parietal lobe and right intracalcarine cortex in the occipital lobe. Table S1 shows r and *p_lin_* of all 48 ROIs considered. The results of t-tests performed to compare the significant *m* and *MD_0_* parameters obtained for SZ and HC are shown in Table 5.

**Table 5:** Comparison between linear age-related trajectories of mean diffusivity in SZ and HC subjects.

ROI	Side	t value	
		m	MD_0_
**Frontal lobe**			
Frontal operculum cortex	L	−1.3114	**−5.4526**
Frontal pole	L	0.7286	−0.7124
	R	0.9197	−0.9434
Inferior frontal gyrus, pars opercularis	L	1.0615	−2.3875
	R	1.2260	−1.8381
Middle frontal gyrus	L	1.4522	−0.3008
	R	1.8209	−1.1514
Superior frontal gyrus	L	2.0360	0.8150
	R	1.7812	−0.0863
**Temporal lobe**			
Planum polare	L	−1.2769	**−2.8494**
Superior temporal gyrus, anterior division	R	0.0879	−0.6155
Temporal pole	R	−0.3863	**−2.9930**
**Insula**			
Insular cortex	L	−0.4318	**−5.1407**
**Parietal lobe**			
Precentral gyrus	L	1.8112	0.2221
**Occipital lobe**			
Intracalcarine cortex	R	0.8467	**−5.0098**

Frontal lobe: p_main_effect_<0.005; temporal lobe: p_main_effect_<0.001; insula, parietal and occipital lobe: p_main_effect_<0.05. L = left; R = right; ROI = Region of Interest; MD = Mean Diffusivity. t values rejecting the null hypothesis are highlighted in bold.

**Figure 2:**
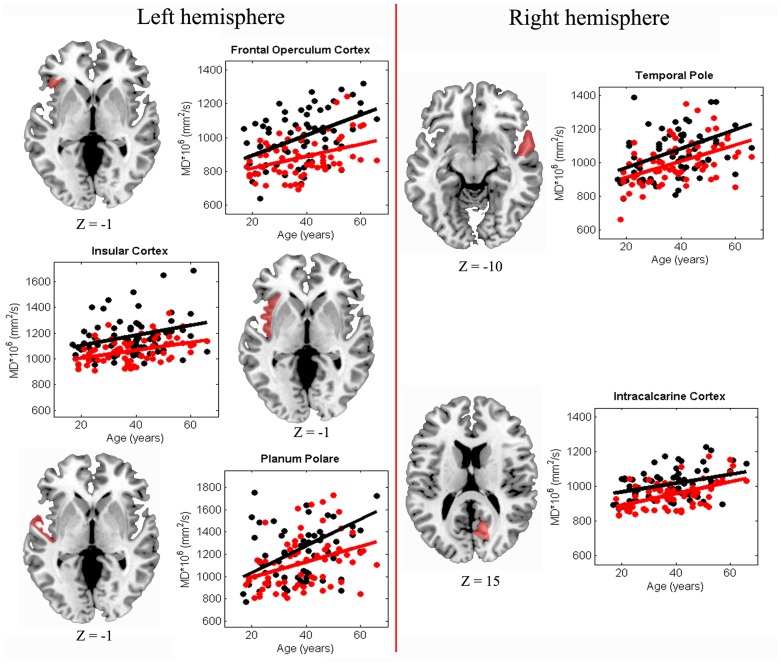
Age-related trajectories of MD within cortical GM of SZ and HC subjects. Scatter plot of MD (* 10^6^ cm^2^/s) as a function of age for SZ (white circles) and HC (red circles). Solid lines represent linear fits for each group. Representative axial slices of the brain are reported for each hemisphere. Cortical regions in which the aging path followed by MD is different in SZ and HC are marked in red. Z coordinates are indicated in MNI space.

The assumption of homogeneity of regression slopes was fulfilled, i.e. no significant diagnosis by age interaction was found. A main effect of diagnosis emerged in the left frontal operculum cortex, left planum polare, right temporal pole, left insula and right calcarine cortex. Figure 2 shows the scatter plots and the relative regression lines of MD as a function of age for SZ and HC in the ROIs where a main effect of diagnosis emerged.

## Discussion

The aim of this study was to investigate age-related changes of brain microstructural parameters in patients affected by SZ and to measure their differences with those of HC subjects. In particular, we focused on: i) age-related trajectories of FA, AD and RD in the WM skeleton, and ii) age-related changes of MD in cortical GM.

Notably, WM microstructure is a classical target of DTI investigations, but the usefulness of DTI metrics in GM has been poorly explored and its interpretation is open to debate [12], [67]–[69]. MD is a valuable parameter for interpreting characteristics regarding the microstructure of isotropic GM, because it provides average spatial information without referring to preferential orientations [37], [68], [69]. To our knowledge, this is the first study on age-related trajectories of MD in cortical GM in SZ. It fills a void in the literature, particularly because cortical GM is already known to be specifically affected by aging in healthy population [36], [70], [71].

Results of our WM analysis revealed a negative relationship between FA and age and between AD and age in both SZ and HC populations, but no relationship between RD and age was found. These FA and AD findings could be explained as due to degradation of WM microtubules [72] and/or to a decline in the number and length of myelinated fibres [34], [73] during aging. However, as these phenomena occur in both SZ and HC groups, the effect of age was independent from the diagnosis. Interestingly, our main finding on WM microstructure is that FA is the only DTI parameter that distinguishes between SZ pathology and HC physiology. Indeed, even if the age-related decrease of FA was indistinguishable between SZ and HC in bilateral WM tracts, including the corona radiata, corpus callosum, thalamic radiations and external capsule, however the FA of SZ patients was permanently reduced from the youngest age and in the entire age range here investigated. Thus, the difference between FA of the two groups remained constant over time. This early WM microstructural brain damage found in patients has already been related to cognitive deficits and psychiatric symptoms in SZ [38], [74]. Various processes may be responsible for the lower WM anisotropy in SZ and caution should be taken before drawing any conclusions [17]. As AD and RD parameters did not discriminate between SZ and HC for WM microstructural age-related changes, it seems highly improbable that the lower FA found early in SZ is due to disruption of axons or to the unfolding of myelin sheaths. The difference should be attributed to the three-dimensional architecture of WM bundles rather than the structure of a single fiber. In fact, the voxel dimension of the processed skeleton maps is three orders of magnitude higher than the section of a single myelinated fibre, thus the measured anisotropy may also be influenced by the spatial distribution of fibres [24], [41]. Moreover, the FA impairment found in SZ appears early in life and remains constant during illness progression. Therefore, this early and enduring WM abnormality may be due to perturbed neurodevelopmental processes (e.g. bacterial and viral infection, oxygen deprivation or excess, and/or elevated systemic proinflammatory cytokine levels, etc.) interfering with normal tract formation [75]. Our results are in line with those reported by Schneidermann et al. [76] but contrast in some way with those of other studies that describe SZ as a disorder characterized by a neurodegenerative process [40], [43]. On the contrary, other studies report that WM microstructural differences between SZ patients and HC are already present by adulthood but tend to diminish with age, suggesting a recovering process [41], [77]. These very contrasting results might be due to different sample sizes [41], neuroimaging methods, age range explored and statistical method adopted to perform analyses [40], [43], [77]. Particularly, Friedman et al. [40] found a greater age-related decrease of WM FA in SZ with respect to HC, but they qualitatively assessed the differences in regression slopes of FA as a function of age in the two diagnostic groups, without referring to any statistical parallelism test. Moreover, Mori et al. [43] investigated progressive changes of WM integrity, as revealed by FA, in SZ by adopting a voxel-based parametric method in the framework of SPM2 (Wellcome Department of Cognitive Neurology, London, UK) [78], an approach that is affected by the use of standard registration algorithms and arbitrariness in the choice of spatial smoothing extent. To cope with these problems, TBSS was specifically designed in later studies to improve the sensitivity, objectivity and interpretability of analysis of multi-subject diffusion imaging studies, and these are reasons why here we adopted this procedure.

Regarding cortical GM, our results are consistent with our subcortical WM findings. Thus, they provide further evidence in support of the pure neurodevelopmental hypothesis (at least in the investigated areas) related to SZ aetiology, and also they help to clarify the localisation of this phenomenon. The age-related increase of MD in SZ and HC was the same in the left frontal operculum cortex, left planum polare, left insular cortex, right temporal pole and right calcarine cortex. However, the MD values in these ROIs were higher in SZ than HC starting from the youngest age and the difference between the two groups remains constant during all the age-range here explored. Interestingly, the cortical GM regions significantly damaged in SZ in our analysis are important trait-marker areas of the disorder [79]–[81]. Since the development of imaging techniques, many studies have highlighted the involvement of frontal and temporal lobes in SZ [6], [38], [82]–[87]. Furthermore, brain abnormalities have been reported across regions involved in linguistic processing [84], [88]. In fact, language disturbance is one of the main clinical features in SZ [89], [90] and language production, in particular, has been shown to be impaired in SZ [91]. Remarkably, the three left fronto-temporal ROIs (i.e. left frontal operculum cortex, left planum polare and left insular cortex) that we found impaired in SZ belong to the Broca’s area, one of the language centres known to be particularly involved in auditory-verbal hallucinations [92]–[94]. Cortical areas of the right hemisphere are also involved in hallucination [95] and this supports our finding that SZ patients are impaired in the right temporal pole. Furthermore, the insula is a brain structure with peculiar features related to its interconnectivity and variety of functions [96], [97]. Because the insula is an important interface between frontal and temporal lobes, some authors have suggested the involvement of the insula in SZ as a consequence of primary frontal and temporal lobe pathologies characteristic of this mental disorder [94], [98], [99]. Other studies have shown that the insula is a key structure in socioemotional simulation or mental emotional representation difficulties that characterize patients affected by SZ [100], [101]. Finally, visual processing deficits are an important pathophysiological feature of SZ [102]–[104], which is coherent with our finding of altered GM microstructure of the visual cortex already present in the early phase of the illness. In light of these considerations, our findings on GM microstructure are very intriguing. In fact, this is the first evidence that microstructural abnormalities of cortical trait-markers regions are already present early in SZ patients’ lives and these alterations remain constant at least until age 65. In any case, further work on GM microstructural age-related trajectories should be carried out to more thoroughly this issue. Particularly, software specifically created and optimized for cerebral cortex, e.g. FreeSurfer [105], [106], should be employed. This could help to more precisely characterize cortical regions and limit errors due to partial volume effect.

Before closing this discussion, some issues that may potentially limit the generalizability/weight of the study results should be mentioned. All of the findings here presented suggest that SZ is a neurodevelopmental disorder. It is known that different brain structures develop at different times [9] and there may be many causes and different timing of the alterations of physiological brain development. It appears that in both WM and GM the microstructural differences between SZ and HC are already present by young adulthood (18 years is the lower limit of the age range here explored). Therefore, although we cannot identify the specific kind of insult that occurred in these patients, we know that it should have happened before this age. One may doubt whether DTI parameters here used are particularly sensitive to the structural consequences of neurodevelopmental errors and less to neurodegenerative processes. Actually, MRI indices here used have been shown to be effective in investigating brain structure in primary neurodegenerative pathologies such as Alzheimer’s and Parkinson’s diseases [26], [107]–[109]. Also, most of the patients here considered were under pharmacological treatment. Although it is not ethical (and therefore practically impossible in the real word) to study untreated SZ patients longitudinally, it could be possible to study them with a cross-sectional method between 18 and 65 years. However, this model should include drug naïve patients with late onset, and therefore with further complications because they who may have different mechanisms compared to classical early onset patients with SZ [110]. Thus, even if ideally we should have investigated age-related changes in brain structure by observing longitudinal changes over time in individual subjects, unfortunately such longitudinal approach involves many logistic complications and requires a great deal of time and financial resources to keep track of people and limit the drop-out rate. These are also the main reasons why no long-term (i.e. more than 10 years) longitudinal studies exist on a large sample of patients with a diagnosis of SZ. Although a cross-sectional design has intrinsic limitations and may involve confounding agents, it allows to perform more realistic investigations on large cohorts of subjects. In our experiment, we opted for this design in order to increase the sample size. The simplicity of linear regression as the model for age-related changes for the whole age range investigated may be seen as a limitation [27], [111]. However, the choice to study subjects aged between 18 and 65 years and the number of subjects considered allowed us to employ the linear approximation [112]. Finally, in the patients evaluated here antipsychotic treatment may have acted protectively, thus preventing the patients’ condition from worsening and keeping the pathological degeneration rate aligned with the physiological one. Many studies in the literature have investigated the effect of medication in brain structural age-related trajectories followed by SZ patients. Most authors focused particularly on DTI microstructural age-related changes in SZ and found no significant effects of medication [43], [77], [113]–[124].

## Conclusion

Our results confirm early microstructural damage in localised brain areas but counter the hypothesis that some type of age-related progression of brain microstructural damage in cortical GM or subcortical WM is present in SZ [116], [117], [121], [125]. The aging process in SZ is still unclear, however, and we are unable to predict with certainty the brain-aging trajectories in individual patients who undergo a MRI exam in the early phase of the disease. The literature on this topic is not homogeneous because the size, age range and medication history of the investigated samples and the methods used to process and analyze structural MRI images strongly affect the results. In future studies a deeper understanding of the issue could be obtained by means of longitudinal evaluations of patients during the illness course.

## Supporting Information

Table S1 Coefficients (and relative plin values) of the correlation between mean diffusivity and age within the cortical regions investigated.
(DOC)
