# Supplementary material for: Cortical Grey Matter and Subcortical White Matter Brain Microstructural Changes in Schizophrenia Are Localised and Age Independent: A Case-Control Diffusion Tensor Imaging Study
Source: PLoS One. 2013 Oct 4;8(10):e75115. doi: 10.1371/journal.pone.0075115 (PMC3790776; doi:10.1371/journal.pone.0075115)
Supplement: Table S1 — Coefficients (and relative plin values) of the correlation between mean diffusivity and age within the cortical regions investigated. (DOC) [file pone.0075115.s001.doc]

| **Supplementary Table S1. Coefficients (and relative p_lin_ values) of the correlation between mean diffusivity and age within the cortical regions investigated** | | | | | |
| --- | --- | --- | --- | --- | --- |
| **ROI** | **Side** | **MD** | | | |
|  |  | **HC** | | **SZ** | |
|  |  | **r** | **p_lin_** | **r** | **p_lin_** |
| **Frontal lobe** |  |  |  |  |  |
| Cingulate gyrus anterior division | L | **0.45** | **<.001** | 0.24 | 0.04 |
|  | R | **0.40** | **<.001** | 0.13 | 0.30 |
| Frontal medial cortex | L | 0.22 | 0.06 | 0.23 | 0.05 |
|  | R | **0.36** | **0.002** | 0.23 | 0.41 |
| Frontal operculum cortex | L | **0.38** | **0.003** | **0.43** | **<.001** |
|  | R | **0.38** | **0.001** | 0.30 | 0.01 |
| Frontal orbital cortex | L | **0.43** | **<.001** | 0.21 | 0.09 |
|  | R | **0.46** | **<.001** | 0.22 | 0.07 |
| Frontal pole | L | **0.48** | **<.001** | **0.37** | **0.002** |
|  | R | **0.54** | **<.001** | **0.42** | **<.001** |
| Inferior frontal gyrus pars opercularis | L | **0.57** | **<.001** | **0.35** | **0.003** |
|  | R | **0.60** | **<.001** | **0.40** | **<.001** |
| Inferior frontal gyrus pars triangularis | L | **0.45** | **<.001** | 0.26 | 0.03 |
|  | R | **0.55** | **<.001** | 0.19 | 0.11 |
| Juxtapositional lobule cortex | L | **0.42** | **<.001** | 0.22 | 0.07 |
|  | R | **0.42** | **<.001** | 0.18 | 0.14 |
| Middle frontal gyrus | L | **0.57** | **<.001** | **0.44** | **<.001** |
|  | R | **0.55** | **<.001** | **0.38** | **<.001** |
| Paracingulate gyrus | L | **0.39** | **<.001** | 0.22 | 0.07 |
|  | R | **0.38** | **0.001** | 0.15 | 0.23 |
| Subcallosal cortex | L | 0.26 | 0.03 | 0.15 | 0.22 |
|  | R | 0.26 | 0.03 | 0.12 | 0.30 |
| Superior frontal gyrus | L | **0.58** | **<.001** | **0.34** | **0.004** |
|  | R | **0.54** | **<.001** | **0.37** | **0.002** |
| **Temporal lobe** |  |  |  |  |  |
| Inferior temporal gyrus anterior division | L | 0.18 | 0.14 | 0.20 | 0.10 |
|  | R | 0.15 | 0.21 | 0.11 | 0.39 |
| Inferior temporal gyrus posterior division | L | 0.19 | 0.11 | 0.10 | 0.42 |
|  | R | 0.07 | 0.59 | 0.11 | 0.38 |
| Inferior temporal gyrus temporoccipital part | L | 0.13 | 0.27 | 0.23 | 0.05 |
|  | R | 0.32 | 0.01 | 0.18 | 0.14 |
| Middle temporal gyrus anterior division | L | 0.24 | 0.04 | 0.13 | 0.27 |
|  | R | 0.21 | 0.09 | **0.36** | **0.003** |
| Middle temporal gyrus posterior division | L | **0.37** | **0.002** | 0.26 | 0.03 |
|  | R | **0.41** | **<.001** | 0.18 | 0.13 |
| Middle temporal gyrus temporoccipital part | L | 0.28 | 0.02 | 0.15 | 0.21 |
|  | R | **0.45** | **<.001** | -0.002 | 0.98 |
| Parahippocampal gyrus anterior division | L | 0.07 | 0.59 | 0.28 | 0.02 |
|  | R | 0.02 | 0.87 | **0.42** | **<.001** |
| Parahippocampal gyrus posterior division | L | 0.21 | 0.09 | 0.19 | 0.11 |
|  | R | 0.19 | 0.12 | 0.31 | 0.008 |
| Planum polare | L | **0.36** | **0.002** | **0.40** | **<.001** |
|  | R | **0.53** | **<.001** | 0.27 | 0.02 |
| Planum temporale | L | **0.44** | **<.001** | 0.28 | 0.02 |
|  | R | **0.61** | **<.001** | 0.16 | 0.19 |
| Superior temporal gyrus anterior division | L | 0.26 | 0.03 | 0.30 | 0.01 |
|  | R | **0.40** | **<.001** | **0.38** | **0.001** |
| Superior temporal gyrus posterior division | L | **0.49** | **<.001** | 0.33 | 0.005 |
|  | R | **0.51** | **<.001** | 0.27 | 0.02 |
| **Supplementary Table S1. Coefficients (and relative p_lin_ values) of the correlation between mean diffusivity and age within the cortical regions investigated (“cont”)** | | | | | |
| **ROI** | **Side** | **MD** | | | |
|  |  | **HC** | | **SZ** | |
|  |  | **R** | **p_lin_** | **R** | **p_lin_** |
| Temporal fusiform cortex anterior division | L | 0.13 | 0.28 | 0.24 | 0.05 |
|  | R | 0.11 | 0.37 | 0.02 | 0.88 |
| Temporal fusiform cortex posterior division | L | 0.11 | 0.37 | 0.23 | 0.06 |
|  | R | 0.10 | 0.39 | -0.03 | 0.81 |
| Temporal occipital fusiform cortex | L | 0.29 | 0.01 | 0.13 | 0.27 |
|  | R | **0.38** | **0.001** | 0.12 | 0.33 |
| Temporal pole | L | 0.34 | 0.004 | 0.34 | 0.004 |
|  | R | **0.46** | **<.001** | **0.36** | **0.002** |
| **Parietal lobe** |  |  |  |  |  |
| Angular gyrus | L | **0.37** | **0.002** | 0.26 | 0.03 |
|  | R | **0.41** | **<.001** | 0.04 | 0.72 |
| Central opercular cortex | L | 0.30 | 0.01 | 0.33 | 0.005 |
|  | R | 0.28 | 0.02 | 0.27 | 0.03 |
| Cingulate gyrus posterior division | L | **0.40** | **<.001** | 0.19 | 0.11 |
|  | R | 0.27 | 0.02 | 0.18 | 0.13 |
| Herschl’ s gyrus | L | **0.48** | **<.001** | 0.34 | 0.004 |
|  | R | **0.57** | **<.001** | 0.19 | 0.12 |
| Lingual gyrus | L | **0.48** | **<.001** | 0.11 | 0.36 |
|  | R | **0.42** | **<.001** | 0.16 | 0.18 |
| Parietal operculum cortex | L | 0.24 | 0.04 | 0.20 | 0.11 |
|  | R | 0.31 | 0.01 | 0.12 | 0.33 |
| Postcentral gyrus | L | **0.52** | **<.001** | 0.25 | 0.04 |
|  | R | **0.53** | **<.001** | 0.28 | 0.02 |
| Precentral gyrus | L | **0.54** | **<.001** | **0.35** | **0.003** |
|  | R | **0.57** | **<.001** | 0.31 | 0.01 |
| Precuneous cortex | L | **0.45** | **<.001** | 0.10 | 0.43 |
|  | R | **0.43** | **<.001** | 0.19 | 0.12 |
| Superior parietal lobule | L | **0.48** | **<.001** | 0.26 | 0.03 |
|  | R | **0.54** | **<.001** | 0.28 | 0.02 |
| Supramarginal gyrus anterior division | L | 0.30 | 0.01 | **0.34** | **0.004** |
|  | R | **0.46** | **<.001** | 0.29 | 0.02 |
| Supramarginal gyrus posterior division | L | 0.34 | 0.005 | 0.19 | 0.11 |
|  | R | **0.39** | **<.001** | 0.15 | 0.20 |
| **Insula** |  |  |  |  |  |
| Insular cortex | L | **0.39** | **0.001** | **0.27** | **0.02** |
|  | R | **0.47** | **<.001** | 0.20 | 0.09 |
| **Occipital lobe** |  |  |  |  |  |
| Cuneal cortex | L | **0.40** | **<.001** | 0.13 | 0.28 |
|  | R | **0.41** | **<.001** | 0.14 | 0.24 |
| Intracalcarine cortex | L | **0.53** | **<.001** | 0.27 | 0.02 |
|  | R | **0.53** | **<.001** | **0.37** | **0.002** |
| Lateral occipital cortex inferior division | L | 0.30 | 0.01 | 0.22 | 0.06 |
|  | R | **0.47** | **<.001** | 0.003 | 0.98 |
| Lateral occipital cortex superior division | L | **0.48** | **<.001** | 0.21 | 0.08 |
|  | R | **0.59** | **<.001** | 0.08 | 0.49 |
| Occipital fusiform gyrus | L | **0.38** | **0.001** | 0.10 | 0.42 |
|  | R | **0.42** | **<.001** | 0.13 | 0.28 |
| Occipital pole | L | **0.48** | **<.001** | 0.16 | 0.18 |
|  | R | **0.51** | **<.001** | 0.22 | 0.07 |
| Supracalcarine cortex | L | **0.36** | **0.003** | 0.20 | 0.09 |
|  | R | 0.20 | 0.09 | 0.31 | 0.01 |

Parameters indicating statistically significant correlations are highlighted in bold (Bonferroni correction; frontal and parietal lobes: p_lin_<0.05/12; temporal lobe: p_lin_<0.05/16; insula: p_lin_<0.05; occipital lobe: p_lin_<0.05/7).
